# Functionalization and metathesis polymerization induced self-assembly of an alternating copolymer into giant vesicles†

**DOI:** 10.1039/d1ra00835h

**Published:** 2021-04-22

**Authors:** Wei Song, Jiamin Shen, Xiang Li

**Affiliations:** Department of Polymer and Composite Material, School of Materials Engineering, Yancheng Institute of Technology Yancheng 224051 China sw121092@ycit.cn +86-0515-8829-8872

## Abstract

A facile fabrication of spherical vesicles and micelles by acyclic diene metathesis (ADMET) polymerization and alternative metathesis polymerization (ALTMET) was investigated. We utilize fluorine (FL) and perylene diimide-based (PDI) α,ω-dienes and α,ω-diacrylates to provide a series of homopolymers and alternating copolymers. When using α,ω-dienes as model monomers, TEM measurement indicates that the aromatic FL and PDI building block induced polymers to generate medium-sized (30–50 nm and 90–120 nm, respectively) micelles and vesicles. It was amazing that alternating copolymers derived from PDI α,ω-dienes and FL α,ω-diacrylates spontaneously form giant vesicles with sizes in the range of 0.7 μm to 2.5 μm. The controlled self-assembly of the organic polymer mediated by ADMET and ALTMET techniques avoided extremely annoying post treatment. Therefore, this work establishes a new, versatile synthetic strategy to create nanoparticles having tunable morphologies with potential application as molecular payload delivery vehicles.

## Abstract 2

Fluorine (FL) and perylene diimide-based (PDI) α,ω-dienes and α,ω-diacrylates were used to synthesise a series of homopolymers and alternating copolymers and provide spherical vesicles and micelles by metathesis polymerization.

## Graphical abstract

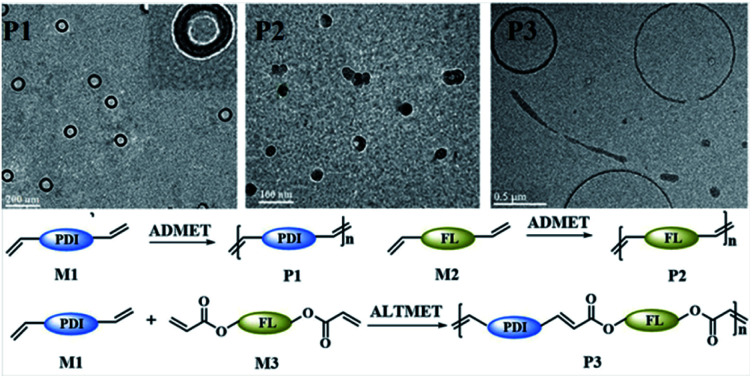

## Introduction

During the past decades, advances in polymer synthesis and macromolecular conjugation reactions have led to progressively complex polymer compositions and architectures being accessible and thus versatile self-assembly techniques have emerged. Self-assembly processes are of particular importance for the formation of defined, monodisperse structures on multiple length scales. Polymerization induced self-assembly (PISA) is an emerging area that couples control of chain-growth and thus dynamic self-assembly morphological versatility, *e.g.*, spherical, cylindrical, and vesicular nanomaterials.^1^ Nevertheless, for access to highly tailorable and readily available nanomaterials, complementary approaches to self-assembly are needed which offer broadened structural versatility to solvophobic blocks and which are value-added for the self-assembly of existing block copolymer substrates.^2–5^ However, PISA is mostly constructed from polymers bearing amphiphilic AB diblocks or ABC triblocks, which requires tedious multistep syntheses and sometimes require the preparations of elaborately designed small molecules and solvent selectivity, and so forth.^6–8^

Acyclic diene metathesis (ADMET) polymerization, on the other hand, has also been considered to be an efficient route for the construction of nanomaterials. Advantages such as using a single type of α,ω-diene monomer, the absence of side reactions and *trans* configuration of the metathetically generated double bonds make ADMET attractive for synthesis of defect-free, high-molecular-weight, all-*trans* polyolefins.^9–15^ Moreover, various α,ω-diene and α,ω-diacrylates comonomers are readily incorporated by ADMET polymerization and alternative metathesis polymerization (ALTMET), affording alternative arrangement of multifunctional monomers along the polymer main chain or side chain. However, in spite of intensive and rather mature research of metathesis polymerization, control of the shape and structure of entirely organic nanoparticles *via* ADMET and ALTMET polymerization is difficult. Until now, only few literature available regarding the formation of nanoparticles *via* ADMET polymerization,^16,17^ where precise control of aromatic branch frequency and identity allowing polyolefins spontaneous assemble into nanotubes and micelles. Meanwhile, most previous researches were focused on the synthesis of homopolymers nanoparticles by ADMET methods. However, the utilization of ADMET and ALTMET techniques to produce self-assemble organic polymers has not been touched.

**Scheme 1:**
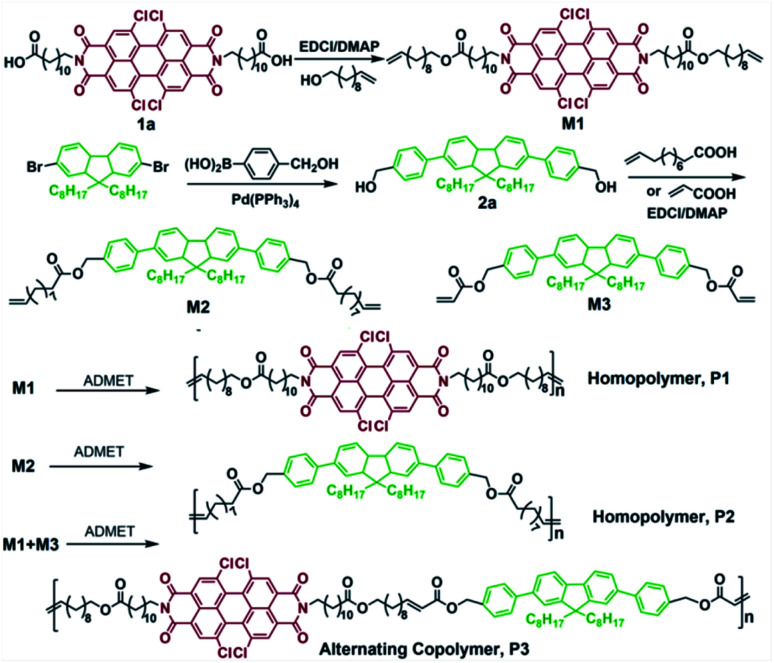
Schematic representation of the synthesis of homopolymers P1, P2 and alternative copolymer P3*via* ADMET and ALTMET polymerization.

In this work, we propose a facile one-pot approach to nanoparticle synthesis, ADMET and ALTMET polymerization-induced self-assembly using chemically and topologically defined patterned monomers as shown in Scheme 1. Precisely spaced aromatic perylene diimide-based (PDI) and fluorine (FL) functional inserts in a constant distance on the polymer backbone is necessary and must be sufficient to provide polymers with curved segments.

ADMET homopolymerization of PDI and FL based α,ω-dienes, ALTMET polymerization between PDI based α,ω-diene and FL based α,ω-diacrylate yield polymers with diverse morphologies. Moreover, these well-designed polymers, with expected tunable morphologies, wide UV-light absorption, higher thermal stability, may find potential application in nanoporous catalyst.

## Experiment

Compound 1a and M1 were synthesized according to literature.^17^ 2,7-Dibromo-9,9-dioctylfluorene and 4-(hydroxymethyl)phenylboronic acid were purchased from Soochiral Chemical Science & Technology Co. Ltd Tetrakis-(triphenylphosphine) palladium(0) Pd(PPh_3_)_4_, ethyl vinyl ether (stabilized with 0.1% *N*,*N*-diethylaniline), and Hoveyda–Grubbs Catalyst 2nd Generation (HG2) and Grubbs 2nd generation catalyst (Ru-II) were obtained from Aldrich. 10-Undecenoic acid, acrylic acid, 1-(3-dimethylaminoprop-yl)-3-ethylcarbodiimidehydrochloride (EDCI·HCl), and 4-dimethylaminopyridine (DMAP) were purchased from Energy Chemical. All reactions were carried out under dry nitrogen atmosphere using standard Schlenk-line techniques. Solvents were distilled over drying agents under nitrogen prior to use. Polymerizations were carried out in Schlenk tubes using of a nitrogen flow to drive off the ethylene condensate for ADMET and ALTMET. ^1^H (300 MHz) and ^13^C (125 MHz) NMR spectra were recorded using tetramethylsilane as an internal standard in CDCl_3_ on a Bruker DPX spectrometer. Melting point was determined by Micro melting point apparatus (Yanoco). UV-vis absorption spectra were measured on a Cary 60 spectrometer. Gel permeation chromatography (GPC) was used to calculate relative number- and weight-average molecular weights (*M*_n_ and *M*_w_) and polydispersity index (PDI) equipped with a Waters 1515 Isocratic HPLC pump, a Waters 2414 refractive index detector, and a set of Waters Styragel columns (7.8 × 300 mm, 5 mm bead size; 10^3^, 10^4^, and 10^5^ Å pore size). The hydrodynamic diameter was determined by means of dynamic light scattering (DLS) analysis using a Malvern Zetasizer Nano-ZS light scattering apparatus (Malvern Instruments, UK) with a He–Ne laser (633 nm, 4 mW). Samples for transmission electron microscopy (TEM) testing were prepared by depositing a drop of the solution (0.05 mg mL^−1^) on a carbon coated Cu grid, and TEM images were recorded on the JEOL2100F microscopes operating at 120 kV. Differential scanning calorimeter (DSC) was performed on a Netzsch 204F1 in nitrogen atmosphere. An indium standard was used for temperature and enthalpy calibrations. All the samples were first heated from 50 to 250 °C and held at this temperature for 3 min to eliminate the thermal history, and then they were cooled to room temperature and heated again from 50 to 250 °C at a heating or cooling rate of 10 °C min^−1^. Thermal gravimetric analysis (TGA) was performed using a SDTA851e/SF/1100 TGA instrument under nitrogen flow from 50 to 800 °C at a heating rate of 10 °C min^−1^.

### Synthesis of 2,7-dihydroxyethylphenyl-9,9-dioctylfluorene (2a)

A mixture of bromo-9,9-dioctylfluorene (3.29 g, 6 mmol), 20% K_2_CO_3_ aqeous solution 40 mL and 4-(Hydroxymethyl)phenylboronic acid (2.19 g, 14.4 mmol) in 60 mL THF was purged with N_2_ for 30 minutes followed by the addition of 350 mg Pd(PPh_3_)_4_. Then the reaction progress proceeded at 80 °C overnight. The resulting mixture was cooled and poured into water, filtrated, and washed with water until the filtrate reached neutrality. After being purified by column chromatography on silica gel using a mixture of petroleum ether/CH_2_Cl_2_ 1 : 1 as eluent, the white powder compound 2a was obtained in 76% yield. ^1^H NMR (CDCl_3_, ppm) (Fig. S1†): *δ* 7.83–7.69 (d, 2H, CCCCH), 7.75–7.67 (m, 4H, CCCH), 7.65–7.57 (m, 4H, CHCH_2_CCH_2_OH), 7.54–7.47 (m, 4H, HOCH_2_CCHCH), 4.85–4.74 (m, 4H, HOCH_2_CCHCH), 2.13–1.99 (m, 4H, CCH_2_CH_2_), 1.89–1.77 (m, 4H, CCH_2_CH_2_), 1.26–1.03 (m, 20H, (CH_2_)_5_CH_3_), 0.87–0.71 (m, 6H, (CH_2_)_5_CH_3_); HR-MS: calcd for C_43_H_58_O_2_Na [M + Na]^+^: 629.9350, found: 629.8379.

### Synthesis of FL-functionalized α,ω-diene monomer (2)

Compound 2a (3.0 g, 5 mmol) was firstly dissolved in 50 mL of anhydrous CH_2_Cl_2_. To this solution, 10-undecenoic acid (2.4 g, 13 mmol), EDCI·HCl (3.0 g, 15.6 mmol) and DMAP (0.1 g, 3 mmol) were added under nitrogen atmosphere in ice-water bath and stirred for 2 h, then the reaction progress proceeded at room temperature and was monitored by TLC. After 4 days, the mixture was washed by dilute hydrochloric acid (5 × 30 mL), followed by water, and dried with anhydrous MgSO_4_. After filtration and removing the solvent, crude product was purified by column chromatography on silica gel using 1 : 5 CH_2_Cl_2_/petroleum ether as eluent. The FL α,ω-diene monomer, M2 was obtained as white waxy solid (4.02 g, yield 86%). ^1^H NMR (CDCl_3_, ppm): *δ* 7.83–7.78 (d, 2H, CCCH), 7.72–7.68 (d, 2H, CCCH), 7.63–7.56 (m, 4H, OCH_2_CCHCH), 7.51–7.45 (d, 4H, OCH_2_CCH), 5.89–5.78 (m, 2H CH_2_=CH), 5.23–5.10 (s, 4H, OCH_2_), 5.05–4.91 (m, 4H, CH_2_=CH), 2.44–2.30 (m, 4H, OCOCH_2_), 2.11–2.00 (t, 4H, CCH_2_), 1.73–1.60 (m, 4H, CH_2_CHCH_2_), 1.45–1.01 (m, 42H, CH_3_(CH_2_)_6_CH_2_C + CH_2_=CHCH_2_CH_2_), 0.84–0.78 (t, 6H, CH_3_CH_2_); ^13^C NMR (CDCl_3_, ppm): *δ* 172.3, 151.8, 141.77, 140.15, 139.58, 134.43, 128.84, 127.39, 126.10, 121.58, 120.04, 80.37, 70.73, 66.64, 55.44, 43.15, 40.37, 31.87, 30.13, 29.30, 23.80, 22.43, 20.07, 14.22; HR-MS: calcd for C_65_H_94_O_4_Na [M + Na]^+^: 961.7152, found: 961.8432.

### Synthesis of FL-functionalized α,ω-diacrylates monomer (M3)

Compound 2a (2.89 g, 4.8 mmol), acrylic acid (1.04 g, 14.4 mmol) and DMAP (0.18 g, 1.44 mmol) were dissolved in CH_2_Cl_2_ (15 mL), and the mixture was stirred at 0 °C for 15 min. EDCI (2.76 g, 14.4 mmol) was then added to the former solution, and stirred for 4 days under nitrogen flow after the solution was warmed to room temperature. The resulting solution was washed three times with deionized water (3 × 80 mL), and the organic layer was dried over anhydrous MgSO_4_. The solvent was then evaporated, and the crude product was purified by chromatographic purification (silica gel, CH_2_Cl_2_/hexane = 1 : 5) to give a viscous colorless solid monomer M3 (2.58 g, 75.8% yield). ^1^H NMR (CDCl_3_, ppm): *δ* 7.79–7.49 (m, 14H, ArH), 6.50–6.46 (d, 2H, CH=CH), 6.23–6.17 (m, 2H, CH=CH), 5.89–5.87 (m, 2H, CH=CH), 5.27 (s, 4H, OCOCH_2_Ar), 2.04–2.01 (m, 4H, CH_2_CCH_2_), 1.43–1.06 (m, 24H, CH_2_), 0.80–0.77 (t, 6H, CH_3_); ^13^C NMR (CDCl_3_), *δ* (ppm): 166.06, 151.69, 141.71, 140.13, 139.49, 134.65, 131.21, 128.83, 128.28, 127.35, 126.01, 121.50, 120.05, 66.14, 55.24, 40.36, 31.74, 29.96, 29.18, 23.77, 22.57, 14.06; HR-MS: calcd for C_49_H_58_O_4_Na [M + Na]^+^: 733.4324; found: 733.9831.

### General procedure for ADMET homopolymerization

A 10 mL of Schlenk tube was charged with M1 or M2 (0.32 mmol) dissolved in 0.7 mL of toluene. In another 10 mL Schlenk tube, Ru-II (10 mg, 12 μmol) was dissolved in 0.3 mL of toluene. After degassed in three freeze–vacuum–thaw cycles, the catalyst solution of Ru-II was then injected into the monomer solution *via* a syringe under vigorous stirring at 60 °C. The reaction was performed for 48 h followed by a slow purge of nitrogen to drive off the ethylene condensate. During this procedure, a second aliquot of the mixture was withdrawn from the tube *via* syringe and 1 mol% Ru-II was added at the predetermined time intervals to monitor the metathesis reaction by GPC, and the resulting mixture gradually became viscous as the molecular weight of polymer increased as well as the solvent toluene partially evaporated. The polymerization was finally quenched by adding excess of ethyl vinyl ether with stirring for another 30 min. The mixture was poured into 20 mL of diethyl ether and the precipitate was isolated by filtration, dried under vacuum at 60 °C to give the unsaturated homopolymer P1 and P2 in high yields.

P1^1^H NMR (CDCl_3_, ppm): *δ* 8.70 (s, pery), 5.28 (s, CH=CH on backbone), 4.26–4.18 (t, COOCH_2_), 4.10–4.01 (t, NCH_2_CH_2_), 2.36–2.17 (t, CH_2_CH_2_COO), 2.14–1.96 (m, CH=CHCH_2_), 1.82–1.22 (m, CH_2_ on alkyl chain); *M*_n_ = 17500, *M*_w_/*M*_n_ = 1.52.

P2^1^H NMR (CDCl_3_, ppm): *δ* 7.83–7.75 (d, CCCH), 7.73–7.65 (d, CCCH), 7.63–7.56 (m, OCH_2_CCHCH), 7.53–7.42 (d, OCH_2_CCH), 5.20 (m, CH=CH on backbone), 5.27–5.14 (s, OCH_2_), 2.49–2.32 (m, OCOCH_2_), 2.11–1.89 (t, CCH_2_), 1.77–1.53 (m, CH_2_CHCH_2_), 1.43–0.99 (m, CH_3_(CH_2_)_6_CH_2_C + CH=CHCH_2_CH_2_), 0.84–0.75 (t, CH_3_CH_2_); *M*_n_ = 16700, *M*_w_/*M*_n_ = 1.52.

#### Representative procedure for preparation of AB-alternating copolymer P3

A Schlenk flask was charged with M1 (0.2 mmol, 250 mg) and M3 (0.2 mmol, 145 mg) under nitrogen, which was degassed with three freeze–vacuum–thaw cycles. A 2 mL aliquot of CH_2_Cl_2_ was added, resulting in a 0.2 M monomer solution. A dosage of 2.5 mg of HG-II was added, and the solution was stirred at 40 °C for 24 h and then approximately 0.5 mL ethyl vinyl ether was added. The solution was precipitated into cold diethyl ether and dried under vacuum.

P3^1^H NMR (CDCl_3_, ppm): *δ* 8.70 (s, pery), 7.82–7.76 (d, CCCH), 7.72–7.68 (d, CCCH), 7.63–7.56 (m, OCH_2_CCHCH), 7.53–7.46 (d, OCH_2_CCH), 7.11–7.02 (m, OCOCH=CH), 5.97–5.84 (m, OCOCH=CH), 4.30–4.17 (t, COOCH_2_), 4.14–4.00 (t, CONCH_2_), 2.37–2.28 (t, CH_2_CH_2_COO), 2.27–0.78 (residual H on alkyl chain); *M*_n_ = 28500, *M*_w_/*M*_n_ = 1.61.

## Results and discussion

### FL functionalized α,ω-diene and α,ω-diacrylates monomers synthesis

Two types of α,ω-diene and α,ω-diacrylates monomers M2 and M3 were designed and synthesized based on PDI, FL motifs and found highly effective in chemical self-assembly. FL-functionalized monomer, M2 and M3 with various numbers of methylene spacers between the eater group and the olefin could be conveniently synthesized by simple Suzuki coupling and esterification reaction (Scheme 1). Firstly, the Suzuki coupling reaction of commercially available starting material 2,7-dibromo-9,9-dioctylfluorene with 4-(hydroxymethyl)phenylboronic acid was accomplished, giving the symmetrical compound, 2a with two reactive hydroxyl groups, and then the predetermined monomers M2 and M3 with aromatic FL and aliphatic alkyl spacer between α,ω-diene and α,ω-diacrylates end groups was easily obtained by subsequent esterification reaction of compound 2a with 10-undecenoic acid and acrylic acid in CH_2_Cl_2_ solution at room temperature, respectively, giving M2 and M3 as the waxy powder in reasonable overall yield over one step. Besides, in order to compare the effects of different aromatic groups on the assembly morphology, and consider the repeatability of the functionalization-induced self-assembly behavior, the PDI-functionalized α,ω-diene monomer, M1 was re-synthesized according to literature.^17^ NMR and HR-MS spectroscopy were employed to confirm the chemical structures of FL functionalized monomers. ^1^H NMR spectra (Fig. S2a and S3†) indicates the presence of four aromatic proton signal peaks on FL motif at 7.79–7.49 ppm and the terminal CH=CH protons at both 5.89–5.78 and 5.05–4.91 ppm for monomer M2, 6.50–6.46 and 5.89–5.87 ppm for monomer M3, respectively. Meanwhile, the integration values of peaks of CH=CH and FL units matched well with theory value, illustrating that the one FL core has been successfully attached to two 10-undecenoic acid and Acrylic acid. Furthermore, the molecular weight of monomer M2 and monomer M3 by HR-MS analysis was in good accordance with the calculated value. Both of these points assured that M2 and M3 have been successfully synthesized.

### ADMET homopolymerization of PDI functionalized M1 and FL functionalized M2

**Fig. 1:**
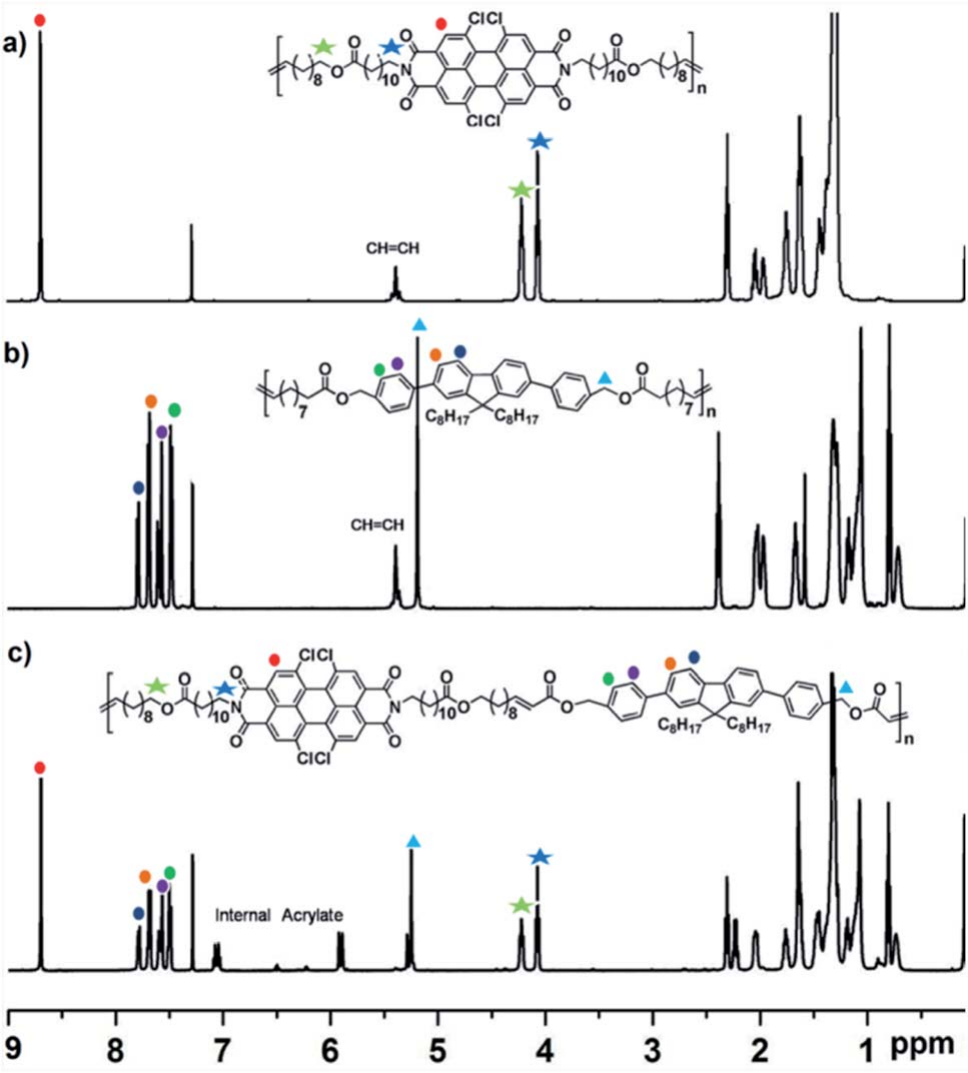
^1^H NMR spectra for (a) P1, (b) P2 and (c) P3 in CDCl_3_.

Motivated by the high functional group tolerance of the Grubbs-type catalysts, we started our investigations with ADMET polycondensation of PDI and FL functionalized α,ω-dienes monomers (M1 and M2) using 1.0 mol% of Grubbs 2nd generation catalyst Ru-II, which was carried out in toluene at 60 °C for 24 h at nitrogen flow in order to remove the byproduct ethylene during this reaction. The reaction mixture became increasingly viscous, after 24 h, it was quenched with ethyl vinyl ether, yielding homopolymers with comparable molecular weight (*M*_n_ = 16700 g mol^−1^ for P1, 17800 g mol^−1^ for P2). Successful polymerization of α,ω-diene monomers can be easily detected from the ^1^H NMR spectra, as the terminal olefin resonances of monomers change into internal alkenes after the polymerization. As shown in Fig. 1b, the signals at 5.89–5.78 ppm 5.05–4.91 ppm of monomer M1 in ^1^H NMR spectrum assigned to the terminal alkenes disappeared after ADMET polymerization, while the protons of newly formed internal alkenes on P1 appeared at 5.20 ppm. As for P2, similarly to that of P1, the vanishing signals at 5.80–5.65 ppm and 4.95–4.75 ppm is accompanied by the emergying signals at 5.28 ppm after ADMET polymerization.^17^ The ^1^H NMR spectra exactly agree with the expected chain structure. Thus, it seems that FL and PDI defected polyolefins between every 22 and 42 carbons spacer have been successfully synthesized.

### ALTMET copolymerization between PDI functionalized α,ω-diene M1 and FL functionalized α,ω-diacrylates M3

Alternating AB copolymers were synthesized by exploiting the selectivity of the metathesis reaction between α,ω-dienes and α,ω-diacrylates. Unlike standard ADMET polymerizations, the ALTMET copolymerization of dienes and diacrylates does not require high vacuum conditions. Consequently, there is greater flexibility in choosing the solvent for this copolymerization. Most ADMET reactions are done neat or with high boiling solvents. Although this approach has been successful, there is a consensus that dichloromethane is the best solvent for metathesis reactions with Grubbs-type catalysts.^10^ Abbas *et al.* has shown that, among several catalysts screened, Hoveyda–Grubbs second generation catalyst (HG2) is the most active toward the cross-metathesis of terminal olefins with acrylates.^18^ Treatment of a 1 : 1 mixture of monomer M1 (PDI based α,ω-dienes) and monomer M3 (FL based α,ω-diacrylates), polymerization reaction performed at 40 °C and employed a total monomer and HG2 ratio of 100 : 1 resulted in the corresponding alternating copolymers, P3 with the moderate molecular weight, *M*_n_ = 21400 g mol^−1^.

Despite lower temperature and monomer concentration, the yield of copolymer P3 could also reach up to 86% (generally, ther eaction temperature was 60 °C in toluene solvent in our previous ADMET polymerization, and monomer concentration reported in the literatures was 0.5–1.0 M). ^1^H NMR spectra could give a good indication that alternating copolymer P3 structure was formed. As can be seen from Fig. 1c, the signal at 8.70 ppm was attributed to the proton on PDI core (Fig. 1a), and the multiple peaks between 7.82–7.46 ppm were resulted from the aromatic proton on FL moiety (Fig. 1b).

It is noteworthy that olefinic protons for A,B-alternating units have a distinct chemical shift from the starting materials and homocoupled units. That is to say, A,B-alternating units (internal acrylates) produce a doublet of triplet signals at 7.13–7.02 ppm and a doublet at 6.03–5.93 ppm. Meanwhile, no internal alkenes were observed that should be observed at 5.43–5.31 ppm where would be attributed to the homopolymer. Moreover, ^1^H NMR spectra displayed sharp signals also demonstrating the high uniformity of the chemical structure of the copolymer P3. Based on this, the extent of AB alternation (*p*) can be easily calculated by integrating two types of peaks, which showed high degrees of alternation of more than 96%.

### UV-vis absorption spectroscopy

**Fig. 2:**
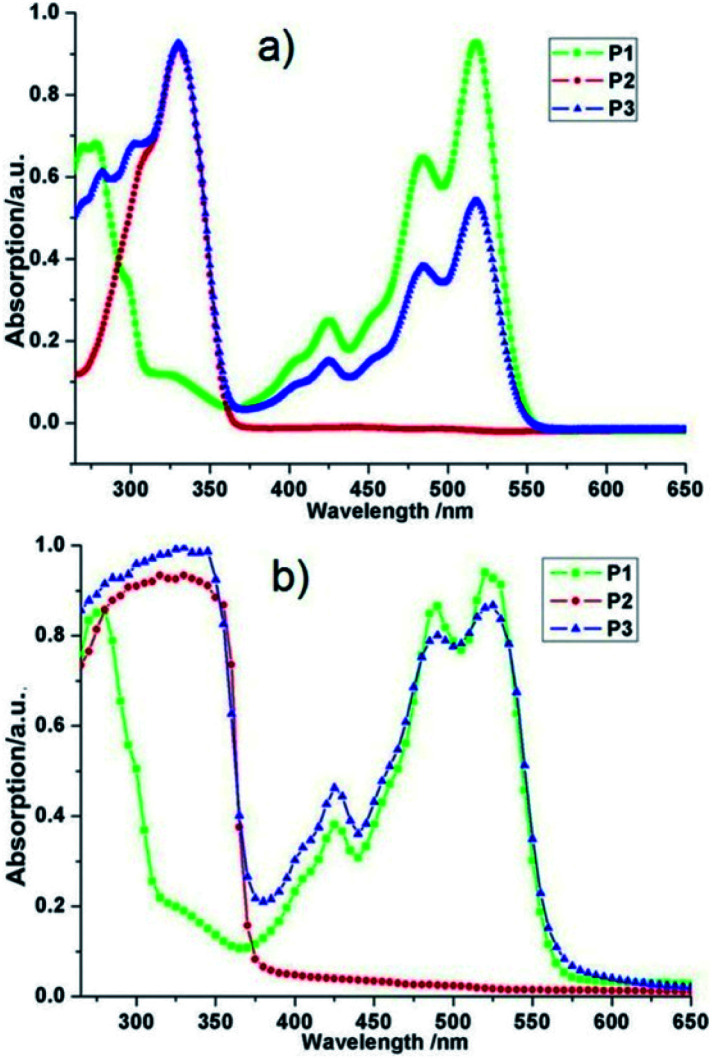
UV-vis spectra for (a) diluted CH_2_Cl_2_ solution of P1, P2, and P3, and (b) in film state of P1, P2, and P3.

Firstly, we characterized the resulting polymers by UV−vis absorption spectroscopy in CH_2_Cl_2_ solvent and observed three characteristic absorption of PDI group at 518 nm, 483 nm, and 424 nm for PDI functionalized homopolymer P1, 330 nm, 300 nm, and 280 nm for FL defected homopolymer P2. As for alternative copolymer P3, obvious two sets of absorption were observed, absorption between 258 and 370 nm corresponding to the PDI moiety and another absorption feature between 376 and 565 nm that represents the conjugated FL unit (Fig. 2a). After drop these three polymer solutions onto quartz glasses, significant enhanced overall intensity was viewed (Fig. 2b); more importantly, the spectrum of copolymer P3 showed another two shoulders at 280 nm and 300 nm which were caused by stronger π–π stacking effect. These results, along with ^1^H NMR analysis revealed the successful synthesis of alternative copolymer P3. All the three polymers have relatively rigid aromatic moieties suggesting that the backbone of polymers had strong π–π stacking effect and more ordered arrangement conformation, which were ideal for self-assembly.

### TEM self-assembled morphology

**Fig. 3:**
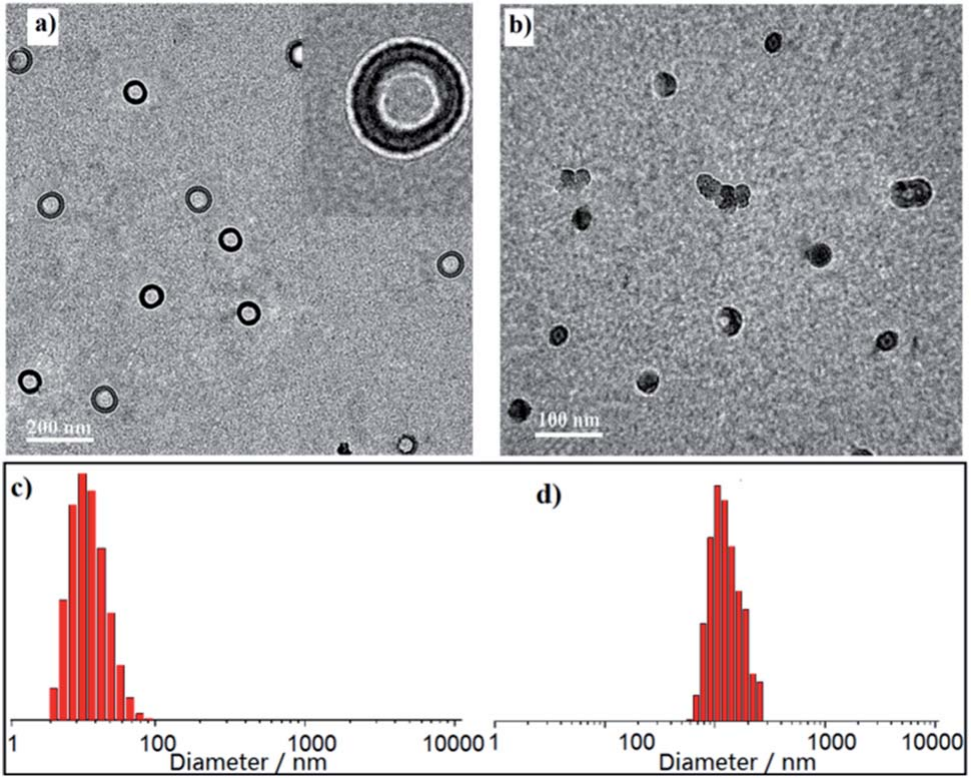
Representative TEM images and DLS data of P1 (a and c) and P2 (b and d) in diluted CH_2_Cl_2_ solutions.

**Fig. 4:**
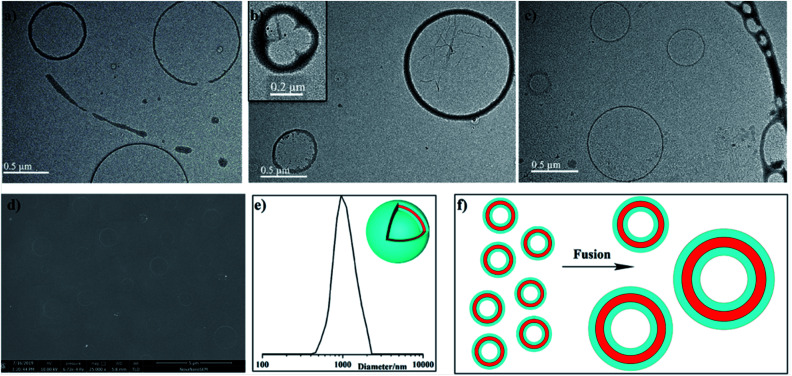
(a) TEM image (a–c), SEM image (d), DLS data (e) of P3 and schematic illustration of shape of giant vesicals.

To check the formation of the nanostructures, the hydrodynamic diameter (*D*_h_) in CH_2_Cl_2_ solution was measured by dynamic light scattering (DLS). Interestingly all the three polymers self-assembled into nanoparticles with sizes mostly in the range of 30–70 nm for P1 (Fig. 3c), 90–120 nm for P2 (Fig. 3d), and 0.9–2.9 μm for P3 (Fig. 4e), respectively. To investigate their nanostructures in detail, we performed transmission electron microscopy (TEM) and observed different kinds of nanoparticles without any post-treatment. Ordered structured homopolymers reported are more prone to assemble into micelles and vesicles.^19–22^ Not surprisingly, PDI functionalized P1 (Fig. 3a) showed uniform vesicles morphology with an average size of 50 nm, and FL inserted P2 (Fig. 3b) exhibited regular spherical particles with an average size of 100 nm. Both in the micelles and vesicles formed by self-assembly the sizes observed in TEM are distinctly lower than described for the DLS results, which possibly be attributed to the shrinkage upon drying.

Surprisingly, embedded alternative PDI–FL building block as a whole repeating unit, copolymer P3 manifested giant sphere vesicles with diameter in the range of 0.7–2.5 μm (Fig. 4a–d). The wall thickness was found to be in nearly 10 nm. A typical fusion of vesicles was quite conforming on the nature of the inter-particle interactions as showing in Fig. 4b and c, the typical fusion of three vesicles was represented in Fig. 4b, which will also continue to collide and actively integrated with one or more larger vesicles. With repeated collision and further induced fusion procession among adjacent vesicles, ultimately forming the giant sphere vesicles morphology that allows the greatest decrease in local free energy relative to the initial ordered structure induced aggregations. It is therefore reasonable to propose that vesicles will experience multiple energy-induced fusions as shown in Fig. 4f with some of this coalescence being sufficiently energetic relief.^23^ These materials of micelles and vesicles can be further utilized to obtain a series of porous nanostructures with tailored sizes and shapes and further loaded with metal catalyst to realize catalytic applications.

### Thermal properties

**Fig. 5:**
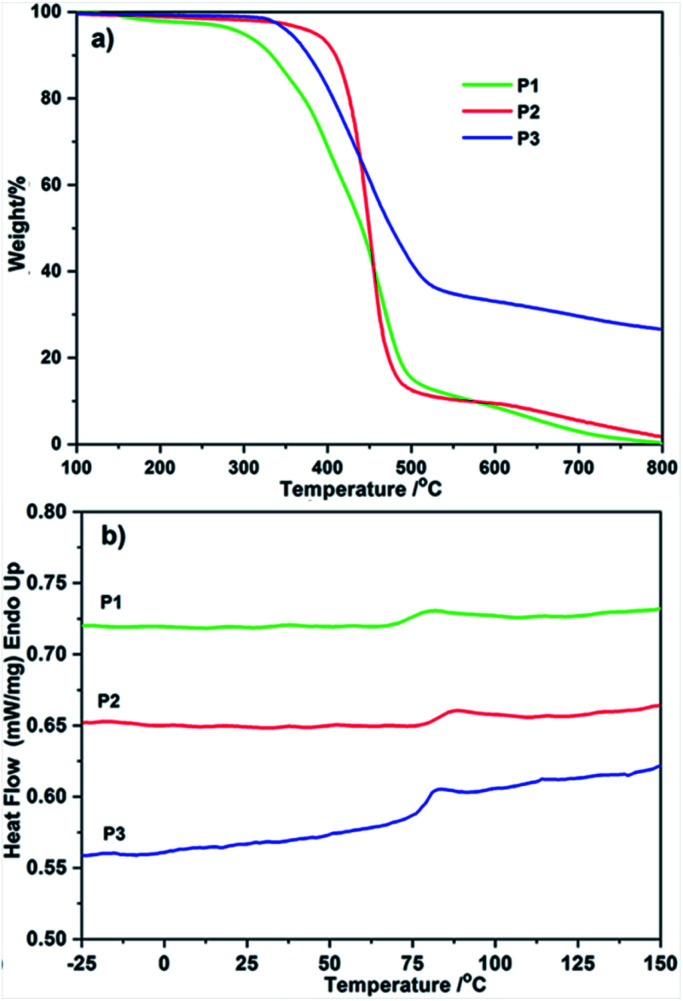
(a) TGA curves and (b) DSC curves for P1, P2 and P3.

The thermal properties of homopolymers P1, P2, and alternative polymer P3 were examined by means of TGA and DSC. In Fig. 5a, the onset temperatures of weight loss (*T*_d_) of P1, P2, and P3 were 298 °C, 386 °C, and 354 °C, respectively. The FL functionalized homopolymers P2 possessed the highest thermal stability than PDI contained P1 and P2, indicating the higher content of benzenes has a positive stimulation to thermal stability. Imported PDI–FL blocks gave P3 a medium decomposition temperature between P1 and P2, the DSC profiles for P1 and P2 show an obvious correlation between steric aromatic defects (PDI and FL moieties) and thermal behavior. As depicted in Fig. 5b, precise incorporation of PDI and FL moieties on polymer backbone generated P1 and P2 with higher *T*_g_ (75 °C and 84 °C), respectively. Similar to that of TGA, a middle *T*_g_ value of P3 (79 °C) was observed and no melting peak was registered for them, a characteristic of amorphous polymers.

## Conclusion

In summary, we have demonstrated a facile synthetic pathway to produce a precision PDI, FL and PDI–FL sequenced polyolefins *via* ADMET polymerization and alternating ADMET polymerization. Imported with alternative PDI, FL and PDI–FL blocks gave polyolefins wider range of light absorption (200–575 nm), higher thermal stability (*T*_d_ 298–354 °C), and lower segmental mobility (*T*_g_ 75–84 °C). Most importantly, it allows facile access to diversified organic polymers having tunable self-assembled morphologies bearing different aromatic functionalities. Polyolefins with precisely placed PDI and FL substituents spaced by long methylene sequences form self-assembled nanoparticals, such as micelles and vesicles varying from tens to hundreds of nanometers. Furthermore, combination of PDI–FL as a whole building block and insertion 25 methylene span between PDI and FL moieties enables the formation of giant sphere vesicles with diameter in the range of 0.7–2.5 μm. Detailed investigations on the role of polymer molecular weight and tailorable chemical composition are in progress for a more comprehensive understanding of the self-assembled behavior. Future work will also be focused on investigating how to cultivate the nanostructures to fulfill catalytic applications.

## Conflicts of interest

The authors declare no conflicts of interest

## Supplementary Material

RA-011-D1RA00835H-s001

## References

[cit1] Lotierzo A., Schofield R. M., Bon S. A. F. (2017). ACS Macro Lett..

[cit2] Howe D. H., Hart J. L., McDaniel R. M., Taheri M. L., Magenau A. J. D. (2018). ACS Macro Lett..

[cit3] Hanisch A., Gröschel A. H., Fortsch M., Drechsler M., Jinnai H., Ruhland T. M., Schacher F. H., Müller A. H. E. (2013). ACS Nano..

[cit4] Man S. k., Wang X., Zhengn J. W., An Z. S. (2020). Chin. J. Polym. Sci..

[cit5] Chen S. L., Shi P. F., Zhang W. Q. (2017). Chin. J. Polym. Sci..

[cit6] Tan J. B., Huang C. D., Liu D. D., Zhang X. C., Bai Y. H., Zhang L. (2016). ACS Macro Lett..

[cit7] Zhang Y., Cao M. J., Han G., Guo T. Y., Ying T. Y., Zhang W. Q. (2018). Macromolecules.

[cit8] Jiang J. H., Zhang X. Y., Fan Z., Du J. Z. (2019). ACS Macro Lett..

[cit9] Schulz M. D., Wagener K. B. (2012). ACS Macro Lett..

[cit10] Le D., Samart C., Kongparakul S., Nomura K. (2019). RSC Adv..

[cit11] Miyashita T., Nomura K. (2016). Macromolecules.

[cit12] Liu X. Q., Chen T. X., Yu F., Shang Y. X., Meng X., Chen Z. R. (2020). Macromolecules.

[cit13] Lv A., Li Z. L., Du F. S., Li Z. C. (2014). Macromolecules.

[cit14] Zhang Z., Qin Y. (2015). ACS Macro Lett..

[cit15] Momcilovic N., Clark P. G., Boydston A. J., Grubbs R. H. (2011). J. Am. Chem. Soc..

[cit16] Jin W. S., Fukushima T., Kosaka A., Niki M., Ishii N., Aida T. (2005). J. Am. Chem. Soc..

[cit17] Song W., You Y., Li T. J., Li J., Ding L. (2018). Chin. J. Polym. Sci..

[cit18] Abbas M., Slugovc C. (2011). Tetrahedron Lett..

[cit19] Changez M., Kang N. G., Lee C. H., Lee J. S. (2010). Small.

[cit20] Changez M., Kang N.-G., Lee J.-S. (2012). Small.

[cit21] Zhu Y., Liu L., Du J. (2013). Macromolecules.

[cit22] Qiu H., Yang Z. N., Köhler M., Ling J., Schacher F. H. (2019). Macromolecules.

[cit23] Anju P., Prasad V. S. (2020). Langmuir.

