# Supplementary material for: Functionalization and metathesis polymerization induced self-assembly of an alternating copolymer into giant vesicles
Source: RSC Adv. 2021 Apr 22;11(25):15153–9. doi: 10.1039/d1ra00835h (PMC8698507; doi:10.1039/d1ra00835h)
Supplement: RA-011-D1RA00835H-s001 [file RA-011-D1RA00835H-s001.pdf]

Electronic Supplementary Material (ESI) for RSC Advances.
This journal is © The Royal Society of Chemistry 2021

Functionalization and Metathesis Polymerization Induced Self-Assembly of Alternating Copolymer into Giant Vesicles

Wei Song*, Jiamin Shen1 and Xiang Li

* *Department of Polymer and Composite Material, School of Materials Engineering, Yancheng Institute of Technology, Yancheng, 224051, China*

**Correspondence: sw121092@ycit.cn (W.S.); Tel.: +86-0515-8829-8872 (W.S.)*

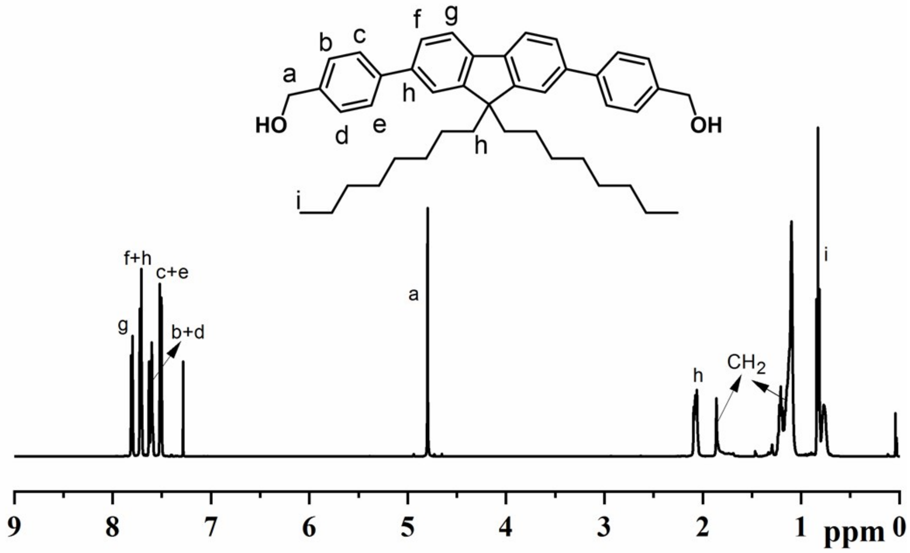

Fig. S1. ^{1}H NMR spectra for **2a** in $CDCl_3$.

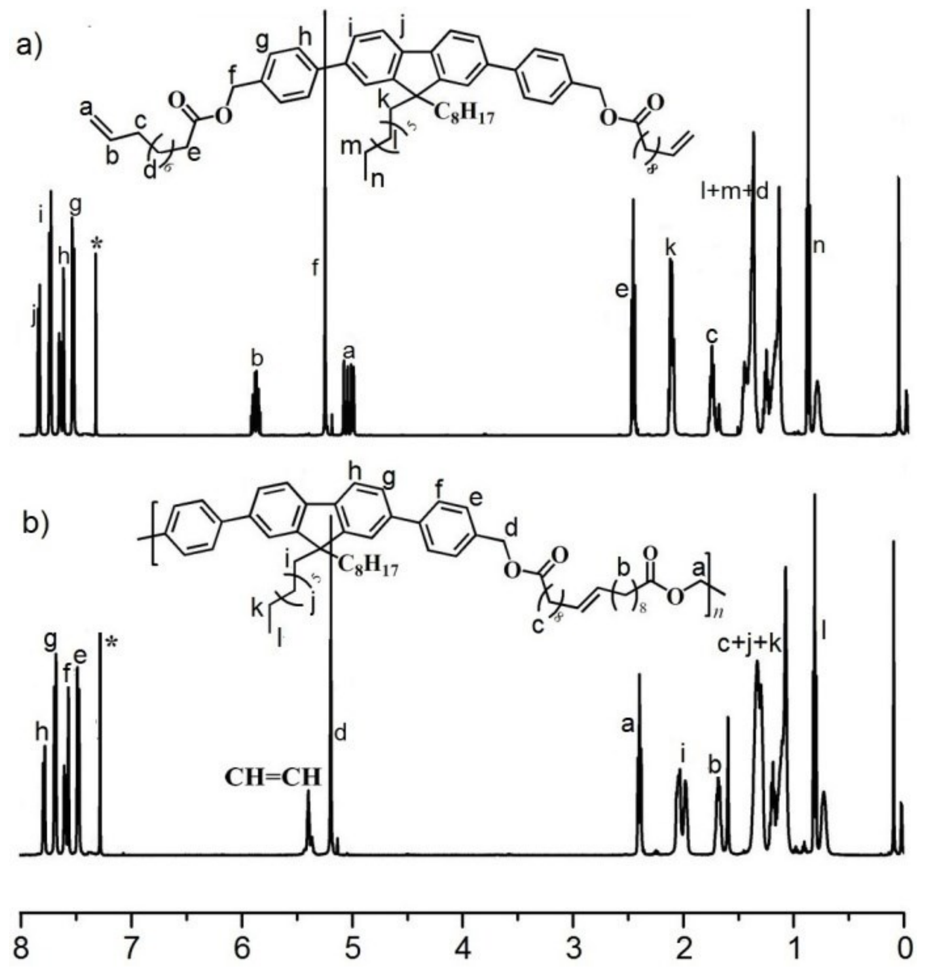

Fig. S2. ¹H NMR spectra for a)monomer **M2** and b) the corresponding polymer **P2** in $CDCl_3$.

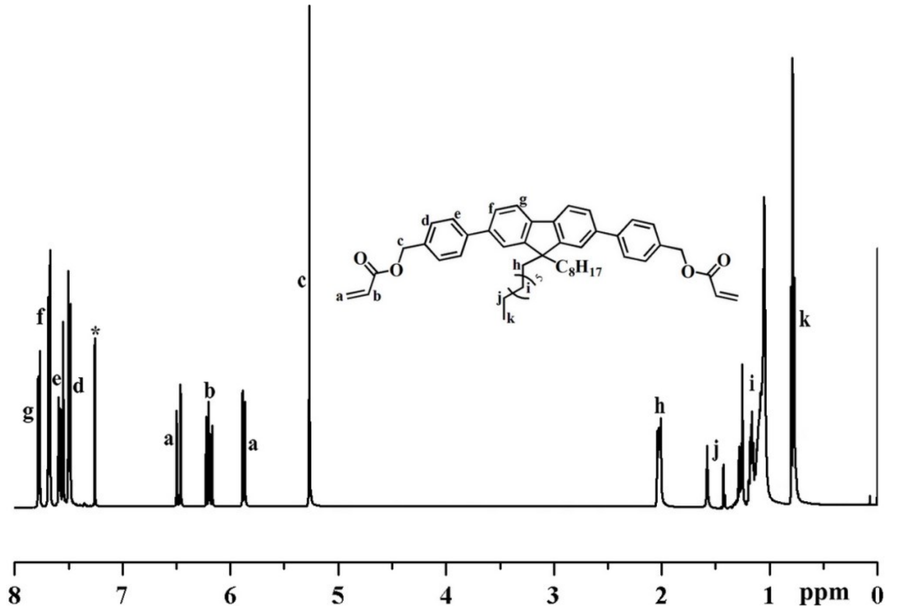

Fig.S3. ¹H NMR spectra for monomer **M3** in $CDCl_3$.
